# Too far to care? A cohort study on travel distance and hospital use in children living with respiratory support

**DOI:** 10.1136/bmjresp-2025-003986

**Published:** 2026-07-14

**Authors:** Johan Florén, Åsa Israelsson-Skogsberg, Magnus Ekström, Berit Lindahl, Agneta Markström, Andreas Palm

**Affiliations:** 1 Faculty of Caring Science, University of Borås, Borås, Sweden; 2 Department of Clinical Sciences, Division of Respiratory Medicine & Allergology, Lund University, Lund, Sweden; 3 Department of Medical Sciences, Respiratory, Allergy and Sleep Research, Uppsala University, Uppsala, Sweden

**Keywords:** Non invasive ventilation, Paediatric Lung Disease, Patient Outcome Assessment, Rare lung diseases, Sleep apnoea, Assisted Ventilation

## Abstract

**Aim:**

Children aged 0–18 years living with respiratory support represent a growing population worldwide. They have complex medical needs and rely on multidisciplinary care. Transportation is often challenging, and paediatric respiratory support teams are concentrated in a limited number of hospitals. As of 2024, Swedish paediatric long-term respiratory care facilities (PRCF) are centralised at four centres. We aimed to explore the relationship between travel distance from home to PRCF, healthcare utilisation and mortality before centralisation.

**Methods:**

A retrospective, population-based cohort study using data from the Swedish Quality Registry for Respiratory Failure (Swedevox) between 2015 and 2021. Multivariable linear regression models and survival analyses were applied to examine the associations between travel distance, healthcare utilisation and mortality.

**Results:**

A total of 596 children were included (mean age 5.4±5.1 years). Of these, 76% lived within 60 min of a PRCF (median 19 km, IQR 8–34), while 24% had travel times exceeding 60 min (median 171 km, IQR 105–268). No association was found between travel time to PRCF and healthcare utilisation or death before age 19. Children whose parents had 12+ years of education underwent more hospitalisations annually (R 0.29, 95% CI 0.01 to 0.57), and children in rural areas had fewer emergency visits (R −0.47, 95% CI 0.84 to −0.10).

**Conclusions:**

No associations were found between travel distance and healthcare utilisation or mortality among children with long-term respiratory support. Centralised PRCF may thus be feasible without compromising outcomes, but reforms should recognise possible unintended effects on children and families.

**WHAT IS ALREADY KNOWN ON THIS TOPIC**

- Children with long-term respiratory support often live far from specialised services, and it is unclear whether travel distance affects their access to care or clinical outcomes.

**WHAT THIS STUDY ADDS**

- This study shows that travel distance to paediatric respiratory care facilities was not associated with healthcare utilisation or mortality, while socioeconomic and geographic factors contributed to differences in healthcare use.

**HOW THIS STUDY MIGHT AFFECT RESEARCH, PRACTICE OR POLICY**

- The findings indicate that centralised paediatric respiratory services may be feasible without harming outcomes, but service planning should account for family burden and socioeconomic disparities.

## Introduction

 Children living with long-term respiratory support are a growing population globally.^1 2^ In this cohort, respiratory support includes continuous positive airway pressure (CPAP); long-term mechanical ventilation (LTMV); high-flow oxygen therapy (HFOT); and phrenic nerve pacing.^3^ In a Swedish context, almost one-third of these children have personal care assistance (PCA).^4 5^ These children also need support from specialised care as they have complex needs requiring multidisciplinary assistance not only for their respiratory support but also for comorbidities.^3 6 7^

Travelling for children with disabilities is described as complicated due to the need for rigorous planning and safety concerns.^8 9^ Delayed care is a potential result of transportation barriers, which are prevalent in groups with poor health or functional limitations.^10^ Studies on paediatric patients requiring surgical care report varied findings. Some have identified increased risks of graft rejection, perforated appendicitis or postoperative mortality associated with rural residence or greater distance to paediatric surgical facilities, while others have found no such associations.^11 12^ Research on adults living in high-income countries has also yielded mixed findings, with some studies reporting adverse health effects related to distance to healthcare facilities, while others do not. The documented health effects include missed appointments, prolonged hospital stays and decreased survival rates.^13^ Studies on the geographic distance to paediatric surgical care have also identified significant socioeconomic disparities in health outcomes.^11^

Sweden is carrying out an ongoing reform aiming for closer and more integrated care on a national level, pinning high hopes on focusing on primary care close to the patient.^14^ Simultaneously, care for children with severe chronic lung diseases will be centralised to four national centres (Skåne University Hospital in Malmö/Lund, Karolinska University Hospital in Stockholm, Norrland University Hospital in Umeå and Sahlgrenska University Hospital in Gothenburg).^15^ The purpose is to generate experience and gain positive effects from accumulated volumes, as cases are scarce and often involve rare diagnoses. This change is in accordance with the European Union’s objective of ensuring access to highly specialised services for rare and complex conditions across member states.^16 17^ Based on its decision-making basis, the National Board of Health and Welfare does not foresee any major negative consequences, and it is perceived that transport will be of the same type as is currently the case, as many children already travel considerable distances. In Sweden, the centralisation of care has been shown to significantly reduce mortality among extremely preterm infants.^18^ Nevertheless, only a limited number of studies, with conflicting results, have examined the effects of increased centralisation on equity in healthcare^19^ or on the consequences for families.^20^ Only one minor study has examined the impact of transportation distance on children living with long-term respiratory support, reporting no significant difference.^21^

The aim of this study was to explore the relationship between transportation distance between home and paediatric long-term respiratory care facilities (PRCF) and the utilisation of healthcare and mortality. We hypothesise that children living further away from PRCF receive less frequent follow-up care, which may increase their need for acute care and hospital stays.

## Methods

### Study design and population

The study used a retrospective population-based cohort design including children aged 0–18 years who had received long-term respiratory support at home for at least 30 days (CPAP, LTMV, HFOT, tracheotomy or phrenic pacing) and were registered in Swedevox between 2015 and 2021. Data were obtained from the Course of DISease reported to the Swedish CPAP Oxygen and VEntilator RegistrY paediatrics (DISCOVERY-P) cohort,^3^ which combines Swedevox information with several national databases, including the National Patient Registry (NPR),^22^ the Total Population Registry,^23^ the Swedish Cause of Death Registry (DORS),^24^ the Longitudinal Integrated Database for Health Insurance and Labour Market Studies (LISA),^25^ the Swedish Intensive Care Registry (SIR)^26^ and the Registry for Interventions under the Act on Support and Service to Certain Disabled Persons (the LSS registry).^27^ The DISCOVERY-P cohort has been detailed elsewhere.^3^

### Outcomes

Six outcomes were evaluated: number of admissions to hospital, number of days spent as an intrahospital patient, number of acute visits to a physician in specialised care, number of planned visits to a physician in specialised care, number of days spent as an intensive care unit (ICU) patient and risk of death. These outcomes are consistent with previous research identifying hospital utilisation and mortality as key indicators in children living with long-term respiratory support.^28^

The number of admissions and the average number of days spent as an intrahospital patient each year were based on data from NPR.^22^ The number of acute visits to physicians and the number of planned visits to physicians were also retrieved from NPR. The number of days spent as an ICU patient each year was retrieved from SIR.^26^ Cases of death (0–18 years) were retrieved from the DORS registry,^24^ and time to death or censoring was obtained from SWEDEVOX,^3^ measured as days utilising respiratory support. Utilisation of healthcare was calculated as an average annual rate for each child during their time in the cohort (annual number of hospital admissions, annual number of days spent in hospital care, annual number of days spent in ICU care and annual number of acute and planned outpatient visits), and was used as a continuous variable.

### Variables

Travel duration from home to PRCF was assessed based on each child’s postcode and reporting PRCF, which were retrieved from the Swedevox registry.^29^ Data on travel duration and distance between home and PRCF were obtained by calculating the shortest car drive between the patient’s postcode and the relevant hospital using Google Maps.^30^ The travel duration was dichotomised as <60 min and ≥60 min. The 60-minute threshold has been used as a benchmark in previous studies on access to care for children,^12^ as well as in several studies concerning adult populations,^13^ as it is considered a meaningful cut-off for assessing timely access to healthcare services.

From Swedevox, information was extracted on participants’ age, sex, mode and duration of ventilatory support, and type of connection (mask or tracheotomy).

Socioeconomic information was taken from the LISA database,^25^ including household income (adjusted for inflation and divided into tertiles^31^) and parental education (categorised as ≤12 years or >12 years of schooling, corresponding to secondary and postsecondary education). Data from the LISA registry were based on the last year before inclusion in the cohort. Country of origin, obtained from the Total Population Register,^23^ was classified as either Swedish-born with at least one Swedish-born parent or Swedish-born/foreign-born with two foreign-born parents.

Data on approved monthly hours of PCA and respite care were retrieved from the LSS registry.^27^ Since these forms of support are frequently used interchangeably, they were combined into one composite variable.^4^ The variable was dichotomised into those using PCA and those without.

Areas of residence were classified as urban or rural, based on categories defined by Statistics Sweden (SCB).^32^ Urban areas comprised large cities (population <200 000), municipalities near large cities (with <40% of the population commuting to the city), medium-sized towns (population <50 000) and municipalities near medium-sized towns (<40% commuting to the town). Rural areas included smaller towns, other urban areas, and rural municipalities with low commuting rates to larger cities.

### Statistical analyses

Associations between travel distance and healthcare utilisation outcomes, and between travel distance and mortality, were evaluated using scatter plot diagrams and crude and multivariate linear regression models. Mortality was analysed using crude and multivariable Cox regression models. Potential confounders included in the models were identified using directed acyclic graphs (www.dagitty.net)^33^ (online supplemental figure S1). Covariates were identified as socioeconomic variables (disposable income, parents’ education and heritage), urban or rural living conditions and age at treatment start.^34^ All analyses were performed in SPSS V.28^35^ and statistical significance was set at p<0.05 (two-tailed).

## Results

**Figure 1:**
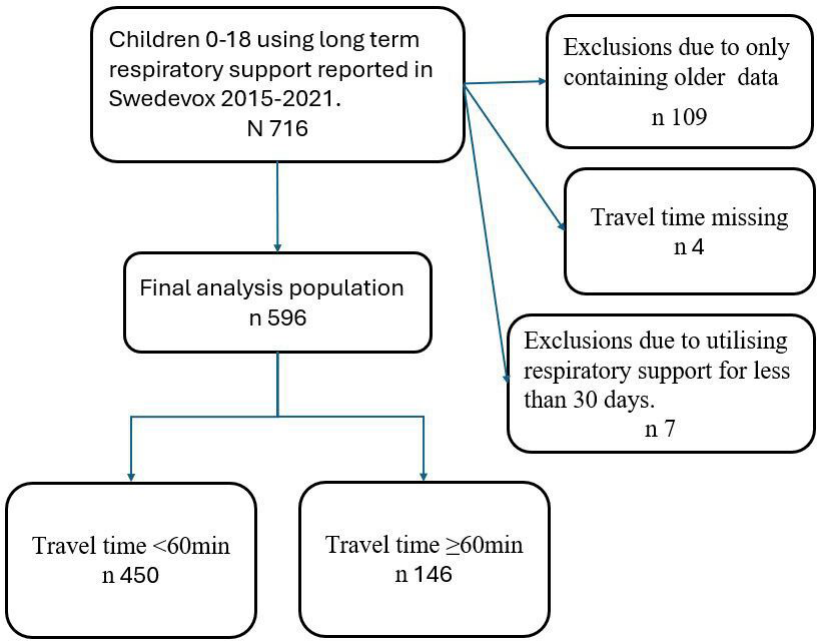
Study flow chart. The figure shows the total number of children assessed for inclusion (N=716), exclusions due to older data, missing travel time or use of respiratory support for less than 30 days and the final analysis population (n=596). Participants are further divided by travel time to paediatric long-term respiratory care facilities: less than 60 min (n=450) and 60 min or more (n=146).

A total of 596 children were included in the analysis (figure 1).

Of the included children, 450 (76%) lived within 60 min of travel duration by car to a PRCF. The median distance from home to a PRCF was 19 km (IQR: 8–34 km) in the <60-minute group and 171 km (IQR: 105–268 km) in the ≥60-minute group. Median follow-up on respiratory support was 1480 days (IQR: 673–2665 days). A vast majority lived in urban areas (table 1).

**Table 1:** Baseline characteristics

Characteristic	All	Travel time <60 min	Travel distance ≥60 min
N	596	450	146
Male sex	337(57)	255 (57)	82 (56)
Age at start, years	5.4±5.1	5.6±5.2	4.8±4.7
0–1 year	213(36)	156 (35)	57 (39)
2–5 years	123(21)	92 (20)	31 (21)
6–10 years	125(21)	92 (20)	33(23)
11–16 years	135(23)	110 (24)	25 (17)
Travel distance (km)*	68±102	24±19	206±127
Travel time (min)*	56±70	25±16	151±86
Utilisation of healthcare			
Annual number of hospital admissions	1.73±1.74	1.69±1.79	1.86±1.58
Annual number of days spent in hospital care	10.5±19.9	10.3±20.8	11.1±16.7
Annual number of acute outpatient visits	0.93±1.22	0.95±1.23	0.89±1.19
Annual number of planned outpatient visits	5.42±4.37	5.61±4.40	4.88±4.22
Annual number of days in ICU	2.08±3.09	1.96±2.98	2.44±3.40
Time using respiratory support (days)	1915±1611	1800±1533	2271±1790
Deceased	70 (12)	51 (11)	19 (13)
Tracheotomy users	78(13)	54 (12)	24 (16)
Type of respiratory support			
CPAP users	197(33)	160 (36)	37 (25)
Long-term ventilator support users	346(58)	243 (54)	103 (70)
Tracheal cannula only/HFOT/phrenic pacing	37(6)	47 (10)	6 (4)
Use of additional treatment (suction, gastric tube, O_2_)	107 (18)	83 (18)	24 (16)
Prescribed duration†			
During sleep (primarily)	449 (85)	350 (86)	99 (82)
Entire day 23–24 hours/totally dependent	65 (12)	45 (11)	20 (16)
Daytime only	13 (3)	11 (3)	2 (2)
Country of origin			
Born in Sweden, one or two native parents	415 (70)	302 (67)	113 (77)
Born in Sweden, two foreign parents/born abroad	181 (30)	148 (33)	33 (23)
Disposable household income (indexed for 2023)‡§	5716±4927	5788±5143	5492±4183
Tertile 1 (lowest income)	195 (33)	140 (31)	55 (38)
Tertile 2 (middle income)	196 (33)	151 (34)	45 (31)
Tertile 3 (highest income)	197 (33)	155 (34)	42 (29)
Parents’ highest educational level¶			
Low, ≤12 years	36 (6)	26 (6)	10 (7)
Medium/low, ≤12 years	218 (37)	159 (35)	59 (40)
High, >12 years	307 (51)	238 (53)	69 (47)
Availability of PCA			
Access to PCA	171 (29)	137 (30)	34 (23)
No access to PCA	425 (71)	313 (70)	112 (77)
Living in rural area	56 (9)	17 (4)	39 (27)

Data are presented as no. (%) or mean±SD.

* 4 missing cases.

† 69 missing cases.

‡ Amount presented in hundreds of Swedish kronor (SEK).

§ 2 missing cases.

¶ 36 missing cases.

CPAP, continuous positive airway pressure; HFOT, high-flow oxygen therapy; ICU, intensive care unit; PCA, personal care assistance.

A total of 20 hospitals report data on children to Swedevox, ranging from a single case to 179 cases (table 2). The geographical distribution of hospitals reporting to Swedevox is shown in online supplemental figure S2.

**Table 2:** Specialised respiratory care facilities reporting data on children to Swedevox

Hospital	Number of children	Average travel distance (km)	Number of children with travel time ≥60 min, n (%)	Average travel duration (min)
Karolinska University Hospital Solna	179	91 (132)	53 (30)	69 (90)
Danderyd Hospital	40	113 (128)	18 (45)	84 (86)
Uppsala University Hospital	34	35 (64)	4 (12)	34 (44)
Mälarsjukhuset Hospital	1	–	1 (100)	–
Vrinnevi Hospital Norrköping	2	7 (1)	0 (0)	10 (1)
Ryhov County Hospital	35	33 (29)	4 (11)	30 (20)
Central Hospital Växjö	13	55 (53)	3 (23)	45 (39)
Blekinge Hospital	9	102 (139)	6 (67)	72 (88)
Skåne University Hospital Malmö	2	143 (198)	1 (50)	64 (54)
Skåne University Hospital Lund	76	68 (77)	21 (28)	64 (55)
Sahlgrenska University Hospital	83	33 (43)	5 (6)	31 (30)
Southern Älvsborg Hospital	24	28 (18)	1 (4)	29 (14)
Northern Älvsborg County Hospital	2	29 (29)	0 (0)	31 (24)
Skaraborg Hospital	3	60 (8)	0 (0)	53 (6)
Central Hospital Karlstad	7	43 (32)	2 (29)	38 (25)
Örebro University Hospital	5	52 (101)	1 (20)	41 (66)
Falun Hospital	1	–	0 (0)	–
Gävle Hospital	9	31 (40)	2 (22)	27 (27)
Sundsvall County Hospital	15	72 (92)	6 (40)	59 (67)
Norrland University Hospital	56	89 (114)	21 (38)	73 (83)

Average (SD)—indicated when only one participant is present.

The associations between travel distance and healthcare utilisation outcomes were negligible (R²≤0.001), indicating that distance explained virtually none of the variation in outcomes. Scatter plots confirmed the absence of any discernible trend (online supplemental figure S3).

In the unadjusted regression models, no associations were found between travel duration ≥60 min and healthcare utilisation. The point estimates varied in direction and magnitude, but no consistent pattern was observed (table 3). When adjusted for confounding factors, no associations were found between travel distance and healthcare utilisation. However, for children whose parents had a higher educational level (>12 years), the average number of hospital admissions per child per year was 0.29 higher compared with those whose parents had a lower educational level. Children in rural areas had 0.47 fewer acute outpatient physician visits per child per year compared with those in urban areas.

**Table 3:** Linear regression models with healthcare utilisation as the dependent variable

	Annual number of admissions as an intrahospital patient		Annual number of days spent as an intrahospital patient		Annual number of days spent as an ICU patient		Annual number of acute outpatient visits to physician		Annual number of planned outpatient visits to physician	
	Crude	Adjusted	Crude	Adjusted	Crude	Adjusted	Crude	Adjusted	Crude	Adjusted
Travel distance										
Travel time <60 min	1	1	1	1	1	1	1	1	1	1
Travel distance ≥60 min	0.17 (–0.16 to 0.50)	0.23 (–0.11 to 0.57)	0.84 (–2.94 to 4.62)	0.97 (–2.97 to 4.92)	0.48 (–0.10 to 1.05)	0.19 (–0.43 to 0.82)	0.06 (–0.29 to 0.18)	0.03 (–0.22 to 0.29)	−0.73 (–1.56 to 0.10)	−0.78 (–1.69 to 0.14)
Country of origin										
Parents born abroad	1	1	1	1	1	1	1	1	1	1
One or two parents born in Sweden	0.13 (–0.18 to 0.44)	−0.11 (–0.42 to 0.21)	−1.33 (–4.83 to 2.16)	−2.57 (–6.24 to 1.09)	−0.22 (–0.76 to 0.32)	−0.41 (–0.99 to 0.17)	0.14 (–0.08 to 0.35)	−0.06 (–0.18 to 0.30)	0.39 (–0.39 to 1.16)	0.10 (–0.75 to 0.95)
Disposable household income										
Tertile 1 (lowest income)	1	1	1	1	1	1	1	1	1	1
Tertile 2 (middle income)	−0.11 (–0.46 to 0.23)	−0.01 (–0.35 to 0.33)	−3.91 (–7.85 to 0.02)	−2.82 (–6.78 to 1.13)	−0.54 (–1.15 to 0.06)	−0.32 (–0.95 to 0.30)	1.15 (−0.85 to 0.40)	0.19 (–0.06 to 0.44)	0.49 (–0.38 to 1.14)	0.55 (–0.36 to 1.46)
Tertile 3 (highest income)	−0.32 (–0.67 to 0.03)	0.03 (–0.38 to 0.33)	−5.05 (–9.00 to −1.11)	−2.07 (–6.19 to 2.05)	−0.63 (–1.24 to −0.27)	−0.06 (–0.71 to 0.59)	−0.38 (–0.28 to 0.21)	0.09 (–0.18 to 0.35)	0.24 (–0.64 to 1.12)	0.50 (–0.50 to 1.45)
Parents’ highest educational level										
Low/medium, ≤12 years	1	1	1	1	1	1	1	1	1	1
High, >12 years	0.27 (–0.03 to 0.56)	0.29 (0.01 to 0.57)	0.69 (–2.61 to 3.99)	1.27 (–2.02 to 4.55)	−0.43 (–0.94 to 0.09)	−0.35 (–0.88 to 0.17)	−0.03 (–0.24 to 0.17)	−0.05 (–0.26 to 0.17)	0.48 (–0.26 to 1.22)	0.38 (–0.38 to 1.14)
Residential classification										
Urban	1	1	1	1	1	1	1	1	1	1
Rural	−0.21 (–0.70 to 0.28)	−0.46 (–0.96 to 0.04)	−0.09 (–5.72 to 5.53)	−1.24 (–7.05 to 4.57)	0.62 (–0.23 to 1.47)	0.52 (–0.39 to 1.43)	−0.40 (–0.74 to −0.05)	−0.47 (–0.84 to −0.10)	−0.57 (–1.81 to 0.67)	−0.34 (–1.68 to 0.99)
Age at start of treatment	−0.12 (–0.15 to −0.09)	−0.12 (–0.15 to −0.09)	−1.05 (–1.35 to −0.75)	−1.02 (–1.34 to −0.69)	−0.14 (–0.18 to −0.09)	−0.13 (–0.19 to −0.08)	−0.04 (–0.06 to −0.19)	−0.04 (–0.06 to −0.02)	−0.12 (–0.19 to −0.05)	−0.13 (–0.21 to −0.06)

Unstandardised coefficients (b) with 95% CIs are reported.

ICU, intensive care unit.

Age was included as a linear covariate in all models. The coefficients varied across outcomes, but the pattern was consistent: younger children had more visits or more hospital days compared with older children. Since the relationships are not entirely linear, the coefficients should be interpreted as average changes across the full age range, with certain ages, particularly the youngest children, having higher numbers of visits or sick days than the average (table 3).

Given the non-normal distribution of residuals for the variables number of days spent as an intrahospital patient, number of days spent as an ICU patient and number of acute visits to a physician, a log transformation was applied as a sensitivity analysis.^36^ Analyses based on log-transformed variables yielded results consistent with those obtained using untransformed data (online supplemental table S2).

No associations were found between travel duration ≥60 min and mortality. Age at start was included for adjustment, but its effect was not interpreted due to limited follow-up among older children (table 4).

**Table 4:** Summary of Cox proportional hazards model of survival analysis based on travel distance

	Unadjusted HR (95% CI)	Adjusted HR (95% CI)
Travel distance		
Travel time <60 min	1	1
Travel distance ≥60 min	0.95 (0.56 to 1.61)	1.10 (0.63 to 1.94)
Country of origin		
Parents born abroad	1	1
One or two parents born in Sweden	0.94 (0.56 to 1.57)	0.93 (0.53 to 1.63)
Disposable household income		
Tertile 1 (lowest income)	1	1
Tertile 2 (middle income)	1.01 (0.58 to 1.76)	1.10 (0.61 to 1.97)
Tertile 3 (highest income)	0.71 (0.40 to 1.27)	0.79 (0.42 to 1.49)
Parents’ highest educational level		
Low/medium, ≤12 years	1	1
High, >12 years	1.04 (0.64 to 1.68)	1.14 (0.69 to 1.88)
Residential classification		
Urban	1	1
Rural	0.71 (0.28 to 1.76)	0.69 (0.26 to 1.78)
Age at start of treatment	0.93 (0.88 to 0.98)	0.93 (0.88 to 0.99)

ICU, intensive care unit.

No associations were found between travel duration and healthcare utilisation when adjusting for availability of PCA in addition to socioeconomic variables (disposable income, parents’ education and heritage), urban or rural living conditions and age at the start of treatment (online supplemental table S3).

## Discussion

In this longitudinal, population-based cohort study, no associations were found between travel time to PRCF and the utilisation of healthcare resources or death before the age of 19. Children with parents who had more than 12 years of education underwent more hospitalisations annually and children residing in rural areas had fewer annual emergency visits to a physician. As one of the largest studies on travel time for children living with respiratory support, the study provides important evidence in a field where assumptions about geographical barriers must be taken into account in planning and financing, but have rarely been tested empirically.

The absence of an association between travel distance and healthcare use may reflect the role of families as a buffer. Parents of children with respiratory support are likely to make the journey to the hospital regardless of whether this is 1 km or 100 km, thereby ensuring access to what they perceive as essential and life-sustaining care.^37^ Parents emphasise that proximity to healthcare services is vital for maintaining a stable and healthy quality of life.^38^ As Andrews^39^ highlights, the significance of a healthcare setting lies less in its physical location than in the quality of care it provides. Families therefore absorb the potential burden of distance through personal sacrifice, prioritising the child’s well-being above all else.^40^,^42^ Families of children living with respiratory support often already manage a demanding everyday life characterised by substantial emotional, physical and social strain.^43^ Against this background, long travel distances may represent an additional burden for families, even if such effects are not captured by measures of healthcare utilisation. In this sense, travel distance may not translate into differences in healthcare utilisation or mortality, since the responsibility for overcoming geographic barriers is largely shifted onto the family. Accordingly, the absence of an association between travel distance and healthcare use should not be interpreted as evidence that distance is without consequences for family life or well-being. From the perspective of family-centred care, families are recognised as key partners in planning and delivering care, yet this central role also entails risks: without sufficient structural and professional support, the heavy reliance on families may compromise their own health and well-being, and in the long run threaten the sustainability of care.^44^

The finding that children with more highly educated parents had more hospital admissions contrasts with a previous large Norwegian study including 1 538 189 children, in which higher parental education was associated with fewer hospital admissions.^45^ One possible explanation for this discrepancy is that although children in socioeconomically disadvantaged families are generally less healthy and at greater risk of hospitalisation,^45 46^ children who live with respiratory support already constitute a medically vulnerable group. In this context, hospital admission is not simply a marker of illness severity, but may also reflect parental capacity to navigate the healthcare system and advocate for care.^47^ Thus, higher parental education may increase the likelihood of hospitalisation when needed, not because of worse health, but due to greater health literacy and system navigation skills. Parents of children with complex care needs often develop substantial knowledge of their child’s condition and may be well placed to recognise early or subtle signs of deterioration.^48^ Higher educational resources may further support this process by facilitating the interpretation of symptoms, communication with healthcare professionals and timely help seeking. In addition, previous research suggests that parental health literacy in childhood chronic illness is associated with knowledge, care management and health-related outcomes, which may also influence how and when families seek hospital-based care.^49^ Therefore, the observed association may reflect differences in parental resources and healthcare navigation rather than differences in the child’s underlying health alone.

Our results also contrasted with a large US-based study, which showed that rural-residing children with medical complexity had more emergency department visits than their urban counterparts.^50^ By contrast, our study found the opposite pattern: children living with respiratory support in rural Sweden had fewer emergency visits. This divergence could reflect cross-country differences in healthcare organisation and access. In rural Sweden, families may benefit from stable care relationships, proactive management and close professional networks that help prevent escalation, as described by physicians working in rural emergency care.^51^ Continuity of care has also been shown to reduce emergency visits and improve coordination for children with medical complexity, underlining why such organisational features are valuable.^52^ However, lower use of emergency care in rural areas may not necessarily indicate better health. It may also reflect barriers to accessing acute care, as long travel distances and transportation challenges can make emergency visits more difficult and may lead families to delay seeking care or rely on other forms of healthcare when possible.^34^ Thus, lower rates of emergency care use in rural Sweden should be interpreted with caution, as they may reflect both organisational strengths and barriers to access rather than lower medical need alone.

## Method discussion

### Strengths

This study benefits from several methodological strengths. Most notably, it draws on a large, nationwide cohort of children receiving respiratory support in Sweden, with data collected from multiple centres. The linkage of clinical data from the Swedevox register with detailed socioeconomic variables from SCB offers a dataset that enables a nuanced analysis of structural and geographic influences on care.^3^

A strength lies in the use of high-quality, government-administered registers to determine outcomes such as healthcare utilisation and mortality. These data sources ensure a high degree of reliability and completeness, contributing to the robustness of the findings.^22^

### Limitations

Although 20 hospitals across the country contributed data, a small number of major hospitals, including one university hospital and five regional or emergency hospitals, did not participate in reporting. However, since children receiving care at these hospitals are not included in the cohort, calculations of travel distance are not affected by their absence. An additional limitation of this study is that data were collected from a total of 20 different hospitals, which may have introduced variability in the consistency and quality of reporting. Differences in data collection protocols, interpretation of criteria, or completeness across sites could potentially affect the reliability of the cohort data. As the NPR does not include visits to primary care, the total number of healthcare contacts may be underestimated. This is particularly relevant if some of the care for this population is provided outside specialist settings. The absence of these data may limit the completeness of the utilisation patterns observed.

While follow-up is nearly complete (93%), some variables, particularly parental education level, have missing data. Children with incomplete background information were more likely to come from single-parent households and immigrant backgrounds, which may correlate with healthcare access and should be considered when interpreting the results.

Another limitation of the study is the limited number of participants in some subgroups, which may have affected the stability and trustworthiness of the statistical results. To strengthen the validity of future findings, studies should strive for larger and more evenly distributed samples.

## Conclusions

No association was found between travel time to PRCF and either healthcare utilisation or mortality. Children whose parents had more than 12 years of education underwent more hospitalisations per year, and children living in rural areas had fewer emergency visits annually. These findings suggest that the ongoing centralisation of paediatric specialised respiratory care may be feasible without compromising clinical outcomes, but reforms should recognise possible unintended effects on children and families. Future research should explore how families living far from healthcare settings experience care, and how rural residence influences their decision to seek acute care.

## Supplementary material

10.1136/bmjresp-2025-003986 online supplemental file 1

## Data Availability

Data are available on reasonable request.
