# Supplementary material for: Too far to care? A cohort study on travel distance and hospital use in children living with respiratory support
Source: BMJ Open Respir Res. 2026 Jul 14;13(1):e003986. doi: 10.1136/bmjresp-2025-003986 (PMC13374413; doi:10.1136/bmjresp-2025-003986)
Supplement: online supplemental file 1 [file bmjresp-13-1-s001.pdf]

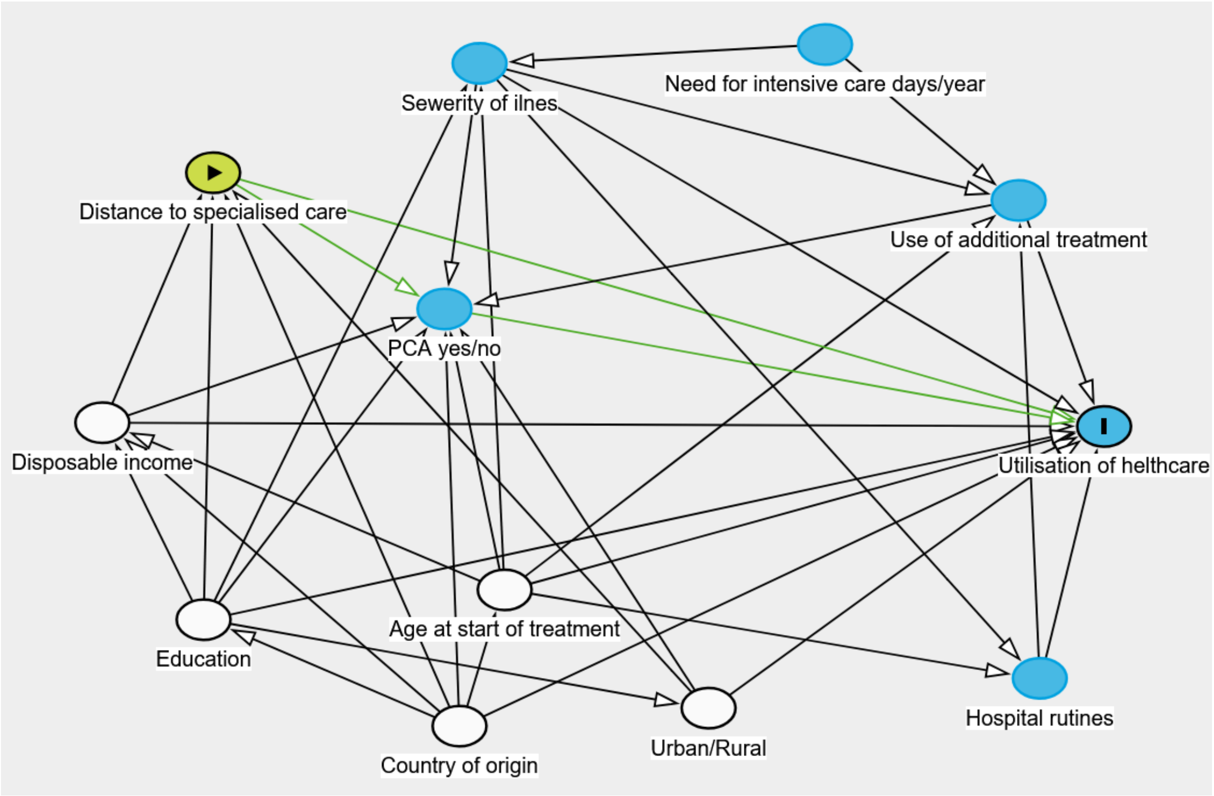

Figure S1. Directed acyclic graph demonstrating the relation between Distance to specialised care and utilisation of healthcare, indicating socioeconomic variables (disposable income, parents' education, and heritage), urban or rural living conditions, and age at treatment start as important covariates.

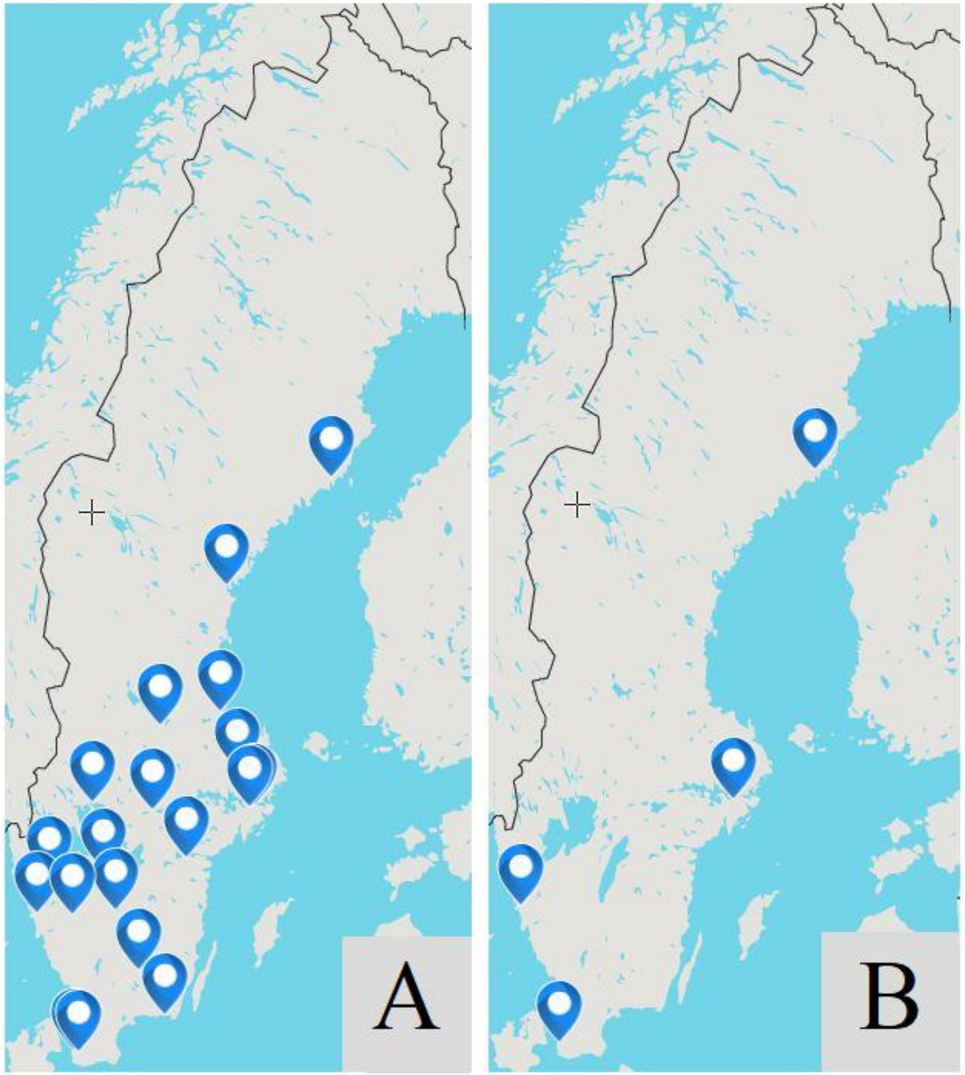

Figure S2. Two maps of Sweden A and B. A shows PRCF reporting to Swedevox before 2024. B shows PRCF reporting to Swedevox after 2024 (www.scribblemaps.com).

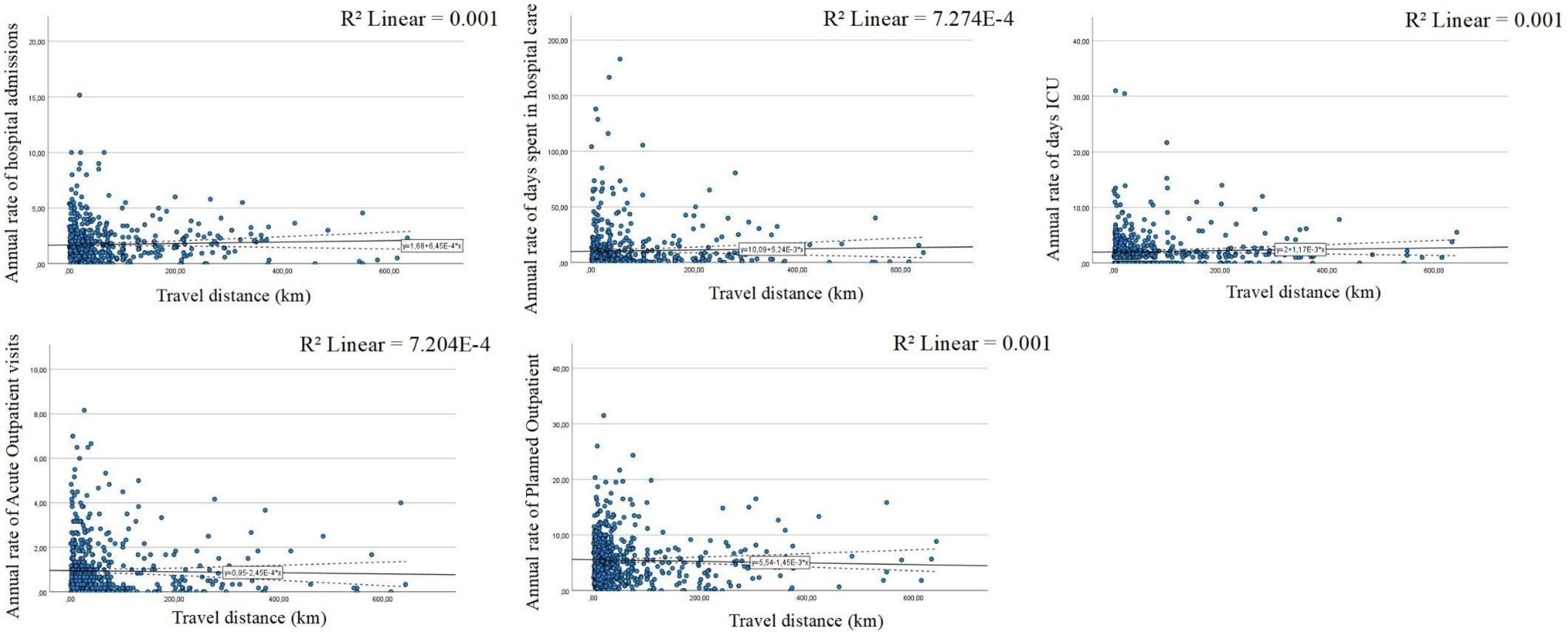

Figure S3. Scatter plots of healthcare utilisation by travel distance. No visible trend was observed, the explained variance was minimal with R^2 at or below 0.001, indicating that travel distance contributed negligibly to variation in healthcare utilisation.

Table S2. Multivariate Linear regression models with utilisation of healthcare as dependent variable, using LOG transformed variables where applicable.

	Annual rate of number of Admissions as an Intrahospital patient		LOG transformed Annual rate of Number of days spent as an Intrahospital patient		LOG transformed Annual rate of number of days spent as an ICU patient		LOG transformed Annual rate of number of Acute Outpatient visits to physician		Annual rate of Planned Outpatient visits to physician	
	Crude	Adjusted	Crude	Adjusted	Crude	Adjusted	Crude	Adjusted	Crude	Adjusted
Travel distance										
Travel time < 60 min	1	1	1	1	1	1	1	1	1	1
Travel distance ≥ 60 min	0.17 (-0.16, 0.50)	0.23 (-0.11, 0.57)	0.18 (-0.10, 0.48)	0.11 (-0.18, 0.41)	0.30 (0.03-0.57)	0.26 (-0.04, 0.57)	0.04 (-0.18, 0.27)	0.19 (-0.04, 0.43)	-0.73 (-1.56, 0.10)	-0.78 (-1.69, 0.14)
Country of origin										
Parents born abroad	1	1	1	1	1	1	1	1	1	1
One or two parents born in Sweden	0,13 (-0.18, 0.44)	-0.11 (-0.42, 0.21)	0.05 (-0.23, 0.32)	-0.04 (-0.32, 0.25)	-0.18 (-0.44, 0.08)	-0.23 (-0.51, 0.053)	0.02 (-0.17, 0.22)	-0.02 (-0.24, 0.19)	0.39 (-0.39, 1.16)	0.10 (-0.75, 0.95)
Disposable household income										
Tertile 1 (lowest income)	1	1	1	1	1	1	1	1	1	1
Tertile 2 (middle income)	0.05 (-0.25, 0.35)	-0.01 (-0.35, 0.33)	-0.06 (-0.32, 0.21)	-0.17 (-0.47, 0.14)	0.03 (-0.26, 0.28)	-0.02 (-0.28, 0.32)	0.14 (-0.05, 0.33)	0.17 (-0.07, 0.40)	0.39 (-0.37, 1.14)	0.55 (-0.36, 1.46)
Tertile 3 (highest income)	-0.26 (-0.56, 0.03	0.03 (-0.38, 0.33)	-0.20 (-0.46, 0.07)	-0.10 (-0.42, 0.22)	-0.15 (-0.40, 0.10)	0.002 (-0.32, 0.32)	-0.13 (-0.32, 0.07)	0.06 (-0.18, 0.30)	0.02 (-0.74, 0.78)	0.50 (-0.50, 1.45)
Parents' highest educational level										
Low/medium, ≤ 12 y	1	1	1	1	1	1	1	1	1	1
High, > 12 y	0.25 (-0.04, 0.54)	0.29 (0.01, 0.57)	0.07 (-0.19, 0.32)	0.09 (-0.18, 0.33)	-0.11 (-0.35, 0.13)	-0.06 (-0.32, 0.20)	-0.10 (-0.29, 0.08)	-0.08 (-0.27, 0.11)	0.46 (-0.28, 1.20)	0.38 (-0.38, 1.14)
Residential classification										
Urban	1	1	1	1	1	1	1	1	1	1
Rural	-0.21 (-0.70, 0.28)	-0.46 (-0.96, 0.04)	0.22 (-0.22, 0,66)	0.09 (-0.36, 0.55)	0.20 (-0.19, 0.60)	0.05 (-0.38, 0.48)	-0.31 (-0.64, 0.03)	-0.43 (-0.80, -0.080)	-0.57 (-1.81, 0.67)	-0.34 (-1.68, 0.99)
Age at start of treatment	-0.12 (-0.15, -0.09)	-0.12 (-0.15, -0.09)	-0.08 (-0.11, -0.06)	-0.08 (-0.11, -0.05)	-0.03 (-0.06, -0.01)	-0.03 (-0.05, -0.003)	-0.03 (-0.05, -0.01)	-0.03 (-0.05, -0.01)	-0.12 (-0.19, -0.05)	-0.13 (-0.21, -0.06)

ICU, intensive care unit.

Table S3. Linear regression models with healthcare utilisation as the dependent variable. Unstandardised coefficients (b) with 95% confidence intervals are reported.

	Annual rate of number of Admissions as an Intrahospital patient		Annual rate of Number of days spent as an Intrahospital patient		Annual rate of number of days spent as an ICU patient		Annual rate of number of Acute Outpatient visits to physician		Annual rate of Planned Outpatient visits to physician	
	Crude	Adjusted	Crude	Adjusted	Crude	Adjusted	Crude	Adjusted	Crude	Adjusted
Travel distance										
Travel time < 60 min	1	1	1	1	1	1	1	1	1	1
Travel distance ≥ 60 min	0.17 (-0.16, 0.50)	0.26 (-0.81, 0.59)	0.84 (-2.94, 4.62)	1.04 (-2.92, 5.00)	0.48 (-0.10, 1.05)	0.23 (-0.40, 0.56)	0.06 (-0.29, 0.18)	0.06 (-0.19, 0.31)	-0.73 (-1.56, 0.10)	-0.62 (-1.51, 0.28)
Country of origin										
Parents born abroad	1	1	1	1	1	1	1	1	1	1
One or two parents born in Sweden	0,13 (-0.18, 0.44)	-0.13 (-0.44, 0.18)	-1.33 (-4.83, 2.16)	-2.62 (-6.29, 1.06)	-0.22 (-0.76, 0.32)	-0.44 (-1.02, 0.14)	0.14 (-0.08, 0.35)	0.04 (-0.20, 0.27)	0.39 (-0.39, 1.16)	-0.01 (-0.85, 0.83)
Disposable household income										
Tertile 1 (lowest income)	1	1	1	1	1	1	1	1	1	1
Tertile 2 (middle income)	-0.11 (-0.46, 0.23)	-0.01 (-0.35, 0.33)	-3.91 (-7.85, 0.02)	-2.83 (-6.78, 1.13)	-0.54 (-1.15, 0.06)	-0.33 (-0.95, 0.30)	1.15 (-.85, 0.40)	0.19 (-0.07, 0.44)	0.49 (-0.38, 1.14)	0.53 (-0.36, 1.43)
Tertile 3 (highest income)	-0.32 (-0.67, 0.03	0.02 (-0.37, 0.33)	-5.05 (-9.00, -1.11)	-2.05 (-6.18, 2.07)	-0.63 (-1.24, -0.27)	-0.05 (-0.70, 0.60)	-0.38 (-0.28, 0.21)	-0.09 (-0.17, 0.36)	0.24 (-0.64, 1.12)	0.53 (-0.41, 1.46)
Parents' highest educational level										
Low/medium, ≤ 12 y	1	1	1	1	1	1	1	1	1	1
High, > 12 y	0.27 (-0.03, 0.56)	0.29 (0.01, 0.57)	0.69 (-2.61, 3.99)	1.26 (-2.03, 4.55)	-0.43 (-0.94, 0.09)	-0.36 (-0.88, 0.16)	-0.03 (-0.24, 0.17)	-0.05 (-0.26, 0.16)	0.48 (-0.26, 1.22)	0.35 (-0.39, 1.09)
Residential classification										
Urban	1	1	1	1	1	1	1	1	1	1
Rural	-0.21 (-0.70, 0.28)	-0.47 (-0.97, 0.02)	-0.09 (-5.72, 5.53)	-1.26 (-7.08, 4.55)	0.62 (-0.23, 1.47)	0.51 (-0.40, 1.42)	-0.40 (-0.74, -0.05)	-0.48 (-0.85, -0.11)	-0.57 (-1.81, 0.67)	-0.40 (-1.71, 0.92)
Age at start of treatment	-0.12 (-0.15, -0.09)	-0.12 (-0.15, -0.09)	-1.05 (-1.35, -0.75)	-1.01 (-1.34, -0.34)	-0.14 (-0.18, -0.09)	-0.13 (-0.19, -0.08)	-0.04 (-0,06, -0.19)	-0.04 (-0,06, -0.18)	-0.12 (-0.19, -0.05)	-0.12 (-0.20, -0.05)
Availability of Personal Care Assistance.	-0.39 (0.07, 0.70)	-0.34 (0.04, 0.64)	-0.12 (-2.45, 4.75)	-0.80 (-2.70, 4.29)	-0.44 (-0.13, -1.00)	-0.42 (-0.14, -0.97)	0.36 (0.14, 0.59)	0.34 (0.11, 0.56)	1.98 (1.19, 2.77)	1.87 (1.08, 2.66)

ICU, intensive care unit.
